# Lumbar endoscopic decompression in the presence of unexpected conjoined nerve root pathology: technical benefits and case illustrations

**DOI:** 10.1093/jscr/rjaf995

**Published:** 2025-12-17

**Authors:** Ralph J Mobbs, Edwin H Y Lui

**Affiliations:** Faculty of Medicine, University of New South Wales, Barker St, Kensington, 2052, NSW, Australia; NeuroSpine Surgery Research Group, Barker St, Randwick, 2031, NSW, Australia; NeuroSpine Clinic, Prince of Wales Private Hospital, Barker St, Randwick, 2031, NSW, Australia; Faculty of Medicine, University of New South Wales, Barker St, Kensington, 2052, NSW, Australia; NeuroSpine Surgery Research Group, Barker St, Randwick, 2031, NSW, Australia

**Keywords:** conjoined nerve root, endoscopic spine surgery, lumbar decompression; anatomical variation

## Abstract

Conjoined nerve roots (CNRs) represent a common yet frequently underdiagnosed congenital anomaly of the lumbar spine that increases the risk of iatrogenic injury. We present two cases of incidentally discovered CNRs successfully managed with full endoscopic spine surgery (ESS). While often missed on preoperative imaging, ESS provides a distinct advantage in managing these variants due to its magnified, high-definition visualization through a minimally invasive approach. This technique allows for the precise identification and safe decompression of unexpected CNRs, serving as both a diagnostic and therapeutic tool. The enhanced clarity and anatomical preservation offered by ESS highlight its vital role in navigating anomalous anatomy, positioning it as a cornerstone for the future of minimally invasive spine surgery.

## Introduction

Conjoined nerve roots (CNRs), a common congenital anomaly of the lumbosacral spine, frequently present significant diagnostic and surgical challenges. A CNR occurs when two adjacent nerve roots share a common dural sleeve or originate from a single foramen, deviating from the typical segmental exit pattern. The reported prevalence varies widely, ranging from 2% to 17% depending on the study design, including cadaveric dissections and imaging series [1–3]. The L5 and S1 nerve roots are the most commonly affected segments [2, 4].

Clinically, the presence of a CNR can mimic or exacerbate radicular symptoms arising from concurrent degenerative pathologies such as disc herniation or lateral recess stenosis, often creating a discrepancy between clinical presentation and imaging findings. Surgically, failure to recognize this anomaly intraoperatively risks iatrogenic nerve injury, incomplete decompression, or persistent postoperative deficits [3, 5, 6]. Although magnetic resonance imaging (MRI) may reveal suggestive features like the ‘corner sign’, preoperative identification remains unreliable, and the definitive diagnosis is most often made during surgery [2, 7].

Safely navigating such anomalous anatomy demands a surgical approach that offers superior visualization and precision. Endoscopic spine surgery (ESS) provides such a platform, combining high-definition magnified optics with fluid-enhanced visualization to allow for detailed delineation of neural structures, minimal tissue disruption, and precise decompression [8–10]. The evolution of ESS from a niche procedure to a central tool in spine care has been driven by its well-documented advantages, a shift described as its transition from the periphery to a pivotal role [11]. This perspective is corroborated by cross-sectional studies of Australian surgeons, which affirm the benefits of ESS regarding improved recovery and patient outcomes, while emphasizing the need for structured training to overcome its technical demands [12].

In this report, we present two cases of lumbar CNRs identified unexpectedly during full endoscopic decompression. These cases highlight the limitations of preoperative diagnostics and demonstrate the technical advantages of ESS in safely and effectively navigating complex, anomalous neural anatomy.

## Case reports

### Case 1

**Figure 1:**
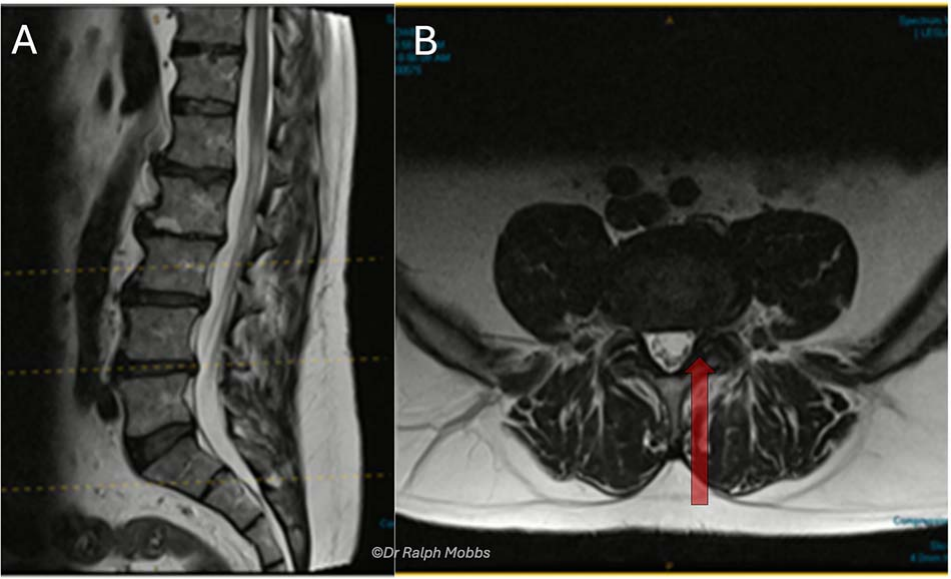
Case 1 mid-sagittal (A) and axial (B) *T*_2_-weighted MRI at L4/5 with lateral recess stenosis (arrow).

A 63-year-old male presented with persistent left-sided L5 radiculopathy that was refractory to conservative management. MRI revealed lateral recess stenosis at the L4–L5 level secondary to facet joint hypertrophy (Fig. 1).

#### Intraoperative findings

**Figure 2:**
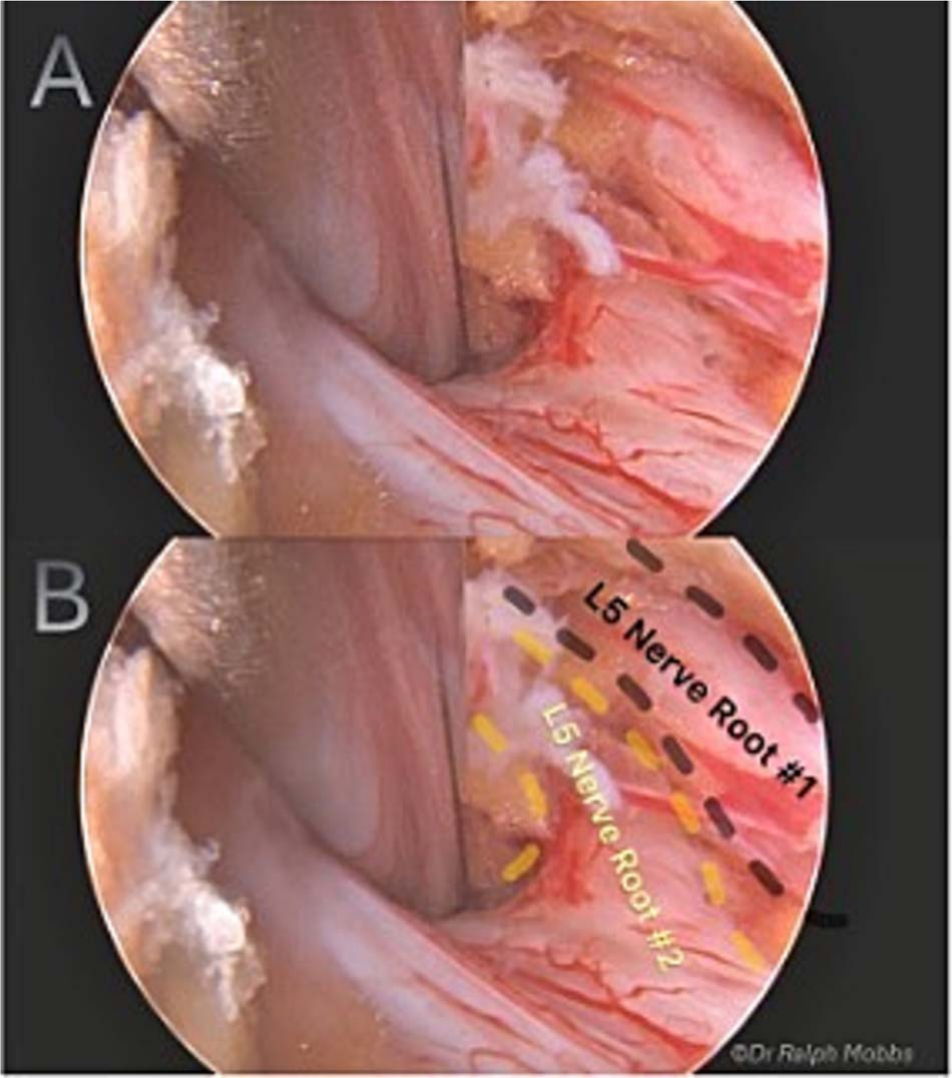
Intraoperative endoscopic view of L5 nerve duplication in the L4/5 lateral recess.

**Figure 3:**
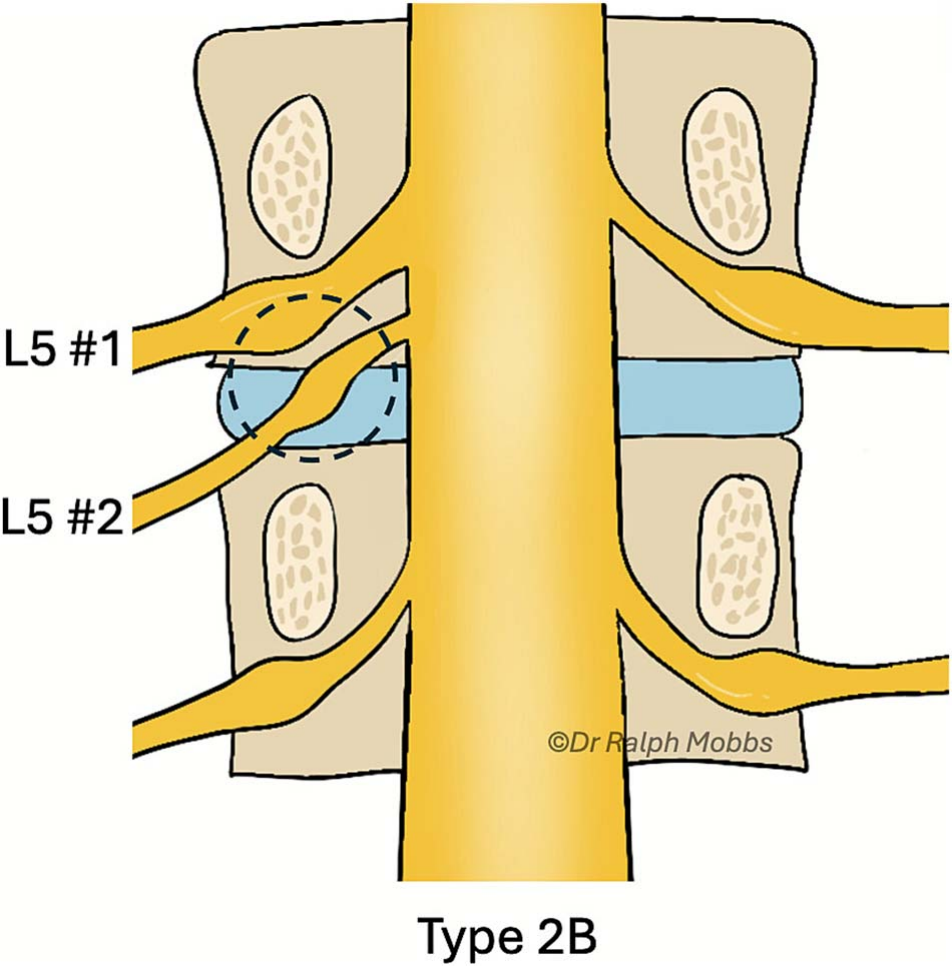
Illustration of the endoscopic view (dashed circle) for case 1, type 2B CNR.

During endoscopic decompression of the left L4–L5 lateral recess, a type 2B conjoined L5 nerve root was identified sharing a common dural sleeve with the adjacent traversing nerve root. The neural elements were meticulously decompressed under direct high-definition visualization (Figs 2 and 3).

#### Postoperative course

The patient experienced mild, transient dysaesthesia, which resolved spontaneously within 2 weeks. At his final follow-up, the preoperative radicular pain had completely resolved, and he had returned to his baseline function without any neurological deficits.

### Case 2

**Figure 4:**
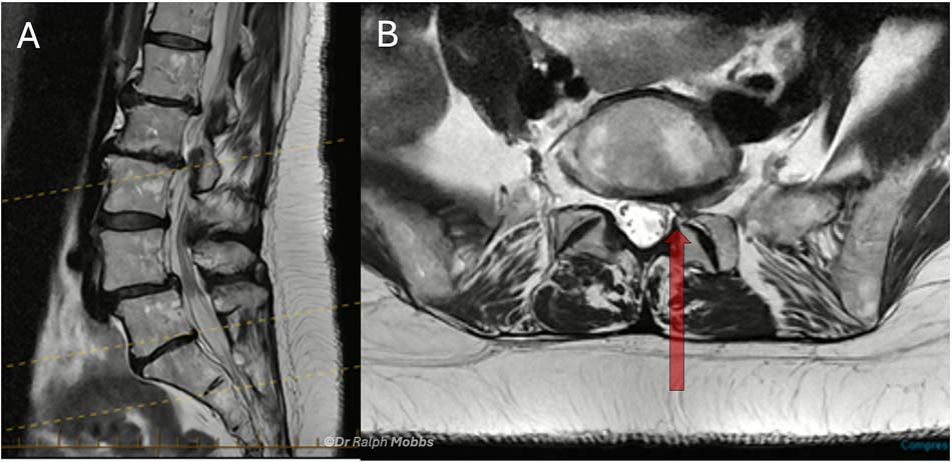
Case 2 mid-sagittal (A) and axial (B) *T*_2_-weighted MRI at L5/S1 with lateral recess stenosis (arrow).

A 60-year-old female presented with a 3-year history of chronic left-sided S1 radiculopathy. MRI demonstrated stenosis of the left lateral recess at L5–S1, with no imaging indication of an underlying nerve root anomaly (Fig. 4).

#### Intraoperative findings

**Figure 5:**
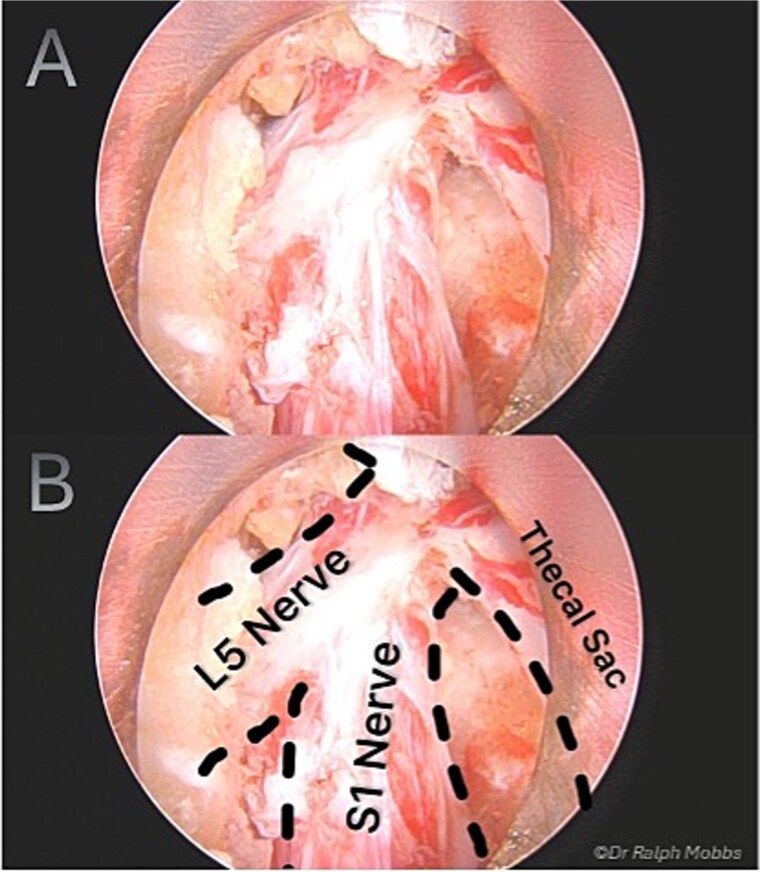
Intraoperative endoscopic view of conjoint L5 and S1 nerve root emerging from a shared dural origin.

**Figure 6:**
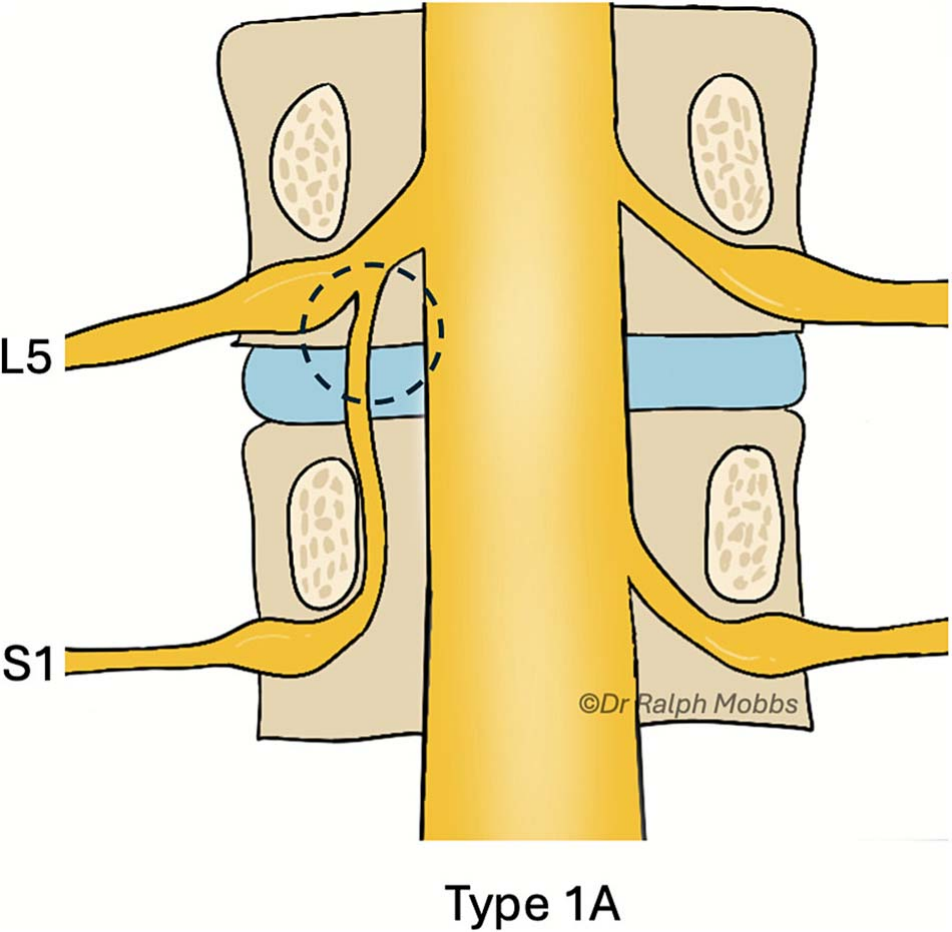
Illustration of the endoscopic view (dashed circle) for case 2, type 1A CNR.

Endoscopic decompression at the left L5–S1 level unexpectedly revealed a type 1A CNR involving both the L5 and S1 roots (Fig. 5), which were found to be emerging from a shared dural origin (Fig. 6).

#### Postoperative course

In the immediate postoperative period, the patient developed a transient partial foot drop (dorsiflexion 4−/5), which fully resolved over 4 weeks. Although she had persistent numbness in the L5 dermatome, her chronic S1 radicular pain resolved completely.

## Discussion

**Figure 7:**
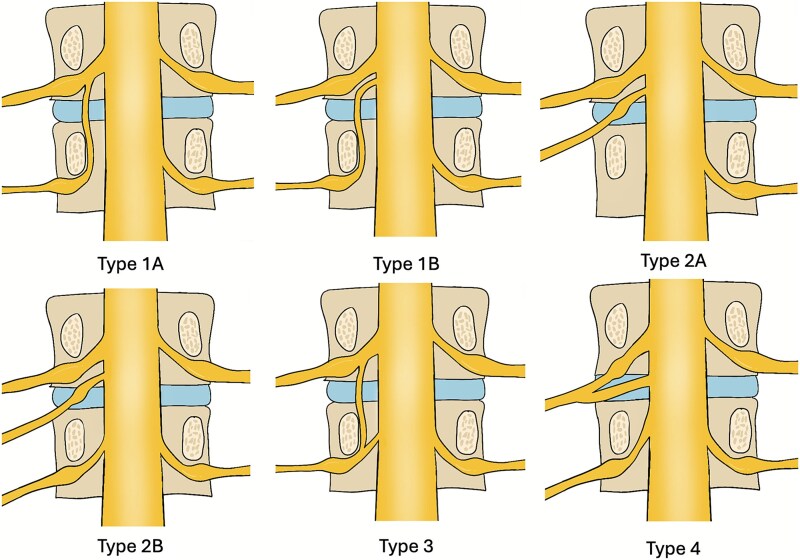
Classification of CNRs by Neidre and Macnab, including type 4 by Burke *et al.* [13, 14].

CNRs continue to pose a significant diagnostic challenge, presenting in several distinct patterns that can complicate spine surgery (Fig. 7). The unreliability of preoperative imaging in identifying this anomaly is a well-documented issue and was evident in our two cases, neither of which had definitive radiological evidence of the CNR [1–3, 7]. The clinical implications of failing to recognize this variation are considerable, potentially leading to incomplete decompression, iatrogenic nerve injury, or persistent postoperative symptoms [5, 6]. These cases reinforce that the surgeon must maintain a high index of suspicion, particularly when a patient’s clinical presentation is discordant with imaging findings.

ESS offers distinct advantages for navigating such anatomical uncertainty. The magnified, high-definition view allows fine dissection and real-time differentiation between neural tissue and surrounding structures, while the continuous fluid irrigation provides meticulous haemostasis, allowing for a clear delineation of the shared dural sleeve from surrounding structures that might otherwise be obscured by bleeding [8–10]. In the confined operative corridor, where critical structures lie in close proximity, this level of clarity is paramount. It enables the surgeon to meticulously trace the nerve root’s course to its origin from the thecal sac, permitting precise identification of any anomalous anatomy. Following identification, a targeted foraminal decompression is performed to ensure the nerve is fully released.

The two cases reported here underscore the practical utility of this approach. In both instances, the endoscope not only facilitated the incidental diagnosis of a CNR but also provided the surgical precision necessary for a safe and effective decompression, resulting in significant pain relief for both patients. It is plausible that without the enhanced visualization and targeted decompression afforded by ESS, a more severe or permanent iatrogenic injury could have occurred. These outcomes demonstrate that the unique features of ESS translate directly into improved intraoperative decision-making when faced with unexpected anatomy.

## Conclusion

CNRs are a common congenital anomaly that can complicate lumbar decompression and increase the risk of iatrogenic injury. As ESS matures, it provides the optical precision and safety margin necessary to both identify and safely manage these neural variants. ESS should therefore be considered not just a minimally invasive alternative, but a platform that expands a surgeon’s diagnostic and therapeutic reach. To further improve outcomes, future research should focus on enhancing preoperative recognition of CNRs through advanced imaging or AI-based analysis and refining surgical training to incorporate anomalous anatomy into endoscopic curricula. With these developments, ESS is poised to become the standard of care for complex lumbar decompressions, ensuring unexpected pathology can be managed with confidence.
